# Modelling environmental DNA transport in rivers reveals highly resolved spatio-temporal biodiversity patterns

**DOI:** 10.1038/s41598-023-35614-6

**Published:** 2023-05-31

**Authors:** Luca Carraro, Rosetta C. Blackman, Florian Altermatt

**Affiliations:** 1 Department of Evolutionary Biology and Environmental Studies, University of Zurich, 8057 Zürich, Switzerland; 2 Department of Aquatic Ecology, Swiss Federal Institute of Aquatic Science and Technology, Eawag, 8600 Dübendorf, Switzerland

**Keywords:** Ecological modelling, Freshwater ecology, Biogeography

## Abstract

The ever-increasing threats to riverine ecosystems call for novel approaches for highly resolved biodiversity assessments across taxonomic groups and spatio-temporal scales. Recent advances in the joint use of environmental DNA (eDNA) data and eDNA transport models in rivers (e.g., eDITH) allow uncovering the full structure of riverine biodiversity, hence elucidating ecosystem processes and supporting conservation measures. We applied eDITH to a metabarcoding dataset covering three taxonomic groups (fish, invertebrates, bacteria) and three seasons for a catchment sampled for eDNA at 73 sites. We upscaled eDNA-based biodiversity predictions to approximately 1900 reaches, and assessed *α*- and *β*-diversity patterns across seasons and taxonomic groups over the whole network. Genus richness predicted by eDITH was generally higher than values from direct eDNA analysis. Both predicted *α*- and *β*-diversity varied depending on season and taxonomic group. Predicted fish *α*-diversity increased downstream in all seasons, while invertebrate and bacteria *α*-diversity either decreased downstream or were unrelated to network position. Spatial *β*-diversity mostly decreased downstream, especially for bacteria. The eDITH model yielded a more refined assessment of freshwater biodiversity as compared to raw eDNA data, both in terms of spatial coverage, diversity patterns and effect of covariates, thus providing a more complete picture of freshwater biodiversity.

## Introduction

Freshwater ecosystems are among the most biodiverse ecosystems worldwide, in relation to their area^1,2^, but also among the most threatened with respect to loss of biodiversity^3,4^. Strategies for conservation of biodiversity should be based on complete biodiversity assessments across spatial and temporal scales, as well as taxonomic groups in order to fully understand and preserve ecosystem functioning^5^. However, this is often not the case for river systems, due to the spatial structure of riverine metacommunities, the coarse spatio-temporal resolution of biodiversity data, a limited taxonomic coverage, and the difficulty to transfer knowledge from one taxonomic group to another^5–8^.

A seminal model for ecological communities in rivers, the river continuum concept^9^, predicted species diversity to have a unimodal patterns in very large rivers (up to 12th Strahler order), with the highest richness observed in mid-order reaches. For most rivers of intermediate size, this translates into an increasing pattern of *α*-diversity in the downstream direction, which has been validated empirically^10^, in particular for fish^11,12^ and macroinvertebrates^13,14^. Conversely, bacteria richness was generally found to follow a decreasing trend in the downstream direction^15–17^. The other component of total (*γ*-) diversity, namely ${\beta}$-diversity, has been less often investigated, although ref.^18^ observed decreasing *β*-diversity with increasing stream size in macroinvertebrates. Such finding is in agreement with the network positioning hypothesis (NPH)^19^, according to which composition of upstream communities is controlled by local environmental factors, while the effect of dispersal processes is more influential in downstream communities, hence reducing *β*-diversity therein. However, universal relationships between biodiversity and stream size appear to be elusive^20^, and following tests of the NPH on diverse river systems and taxonomic groups failed to provide support to this hypothesis^21,22^.

Crucially, most of the studies investigating biodiversity patterns in rivers only focused on specific taxonomic groups, or neglected the temporal dimension of biodiversity, either by considering a snapshot of data collected at a single time point, or by analyzing temporally averaged data^5,8,23,24^. The lack of coherent data on biodiversity across major taxonomic groups is particularly problematic, as it prohibits any systematic comparison of patterns within the same river system and across groups, i.e. covering bacteria, invertebrates and vertebrates at the same time. Such absence of data has been mostly due to the lack of a common method to assess these groups, whereas group-specific methods have traditionally been applied (e.g., electrofishing for fish, kicknetting for macroinvertebrates, biofilm scraping or other sampling for microbes etc.; see ref.^6^). Consequently, most biodiversity studies in riverine systems have been based on spatially scattered, pointwise data limited to a subset of taxonomic groups, hence preventing a spatially highly resolved and/or space filling assessment of biodiversity, as well as an adequate comparison of patterns across taxonomic groups independent of the sampling method chosen.

In this perspective, environmental DNA (eDNA, i.e. DNA isolated from environmental samples^25,26^) has opened new avenues for fast and taxonomically broad biodiversity assessments^27–30^. Environmental DNA samples have been shown to be applicable to a wide, virtually unlimited range of taxonomic groups, and minimally invasive compared to often highly disruptive traditional approaches, such as electrofishing. As such, eDNA techniques are becoming a standard in biodiversity assessments and monitoring, and have the potential to provide unprecedented data also for general ecological studies on the biodiversity and biogeography of organisms^31–34^. In particular, eDNA increases our understanding of biodiversity structure and related ecosystem processes in riverine ecosystems^5^, where eDNA metabarcoding can enhance and complement information on freshwater biodiversity collected from traditional surveys^32,35^. In river networks, due to downstream transportation of DNA molecules with streamflow, eDNA constitutes an aggregated measure of biodiversity across large drainage areas^36–40^. Correct interpretation of eDNA data collected in rivers hence requires consideration of the role of hydrological transport and decay of genetic material. The recently developed eDITH model (*eD*NA *I*ntegrating *T*ransport and *H*ydrology) couples a geomorphological and hydrological characterization of a catchment, eDNA transport and decay dynamics, and a species distribution model, and allows transforming pointwise eDNA data collected at a catchment into predicted maps of taxon density^41–44^. The eDITH model has hitherto been successfully applied to predict the distribution of single species, i.e. a fish parasite and its primary host^41,42^, as well as biodiversity of aquatic insects at a given point in time^43^. Given its generality and the basic assumptions on eDNA shedding and decay processes underpinning its formulation^42^, the eDITH model can in principle be applied to any taxonomic group, and can also be used to identify temporal variations in biodiversity patterns in river networks.

**Figure 1:**
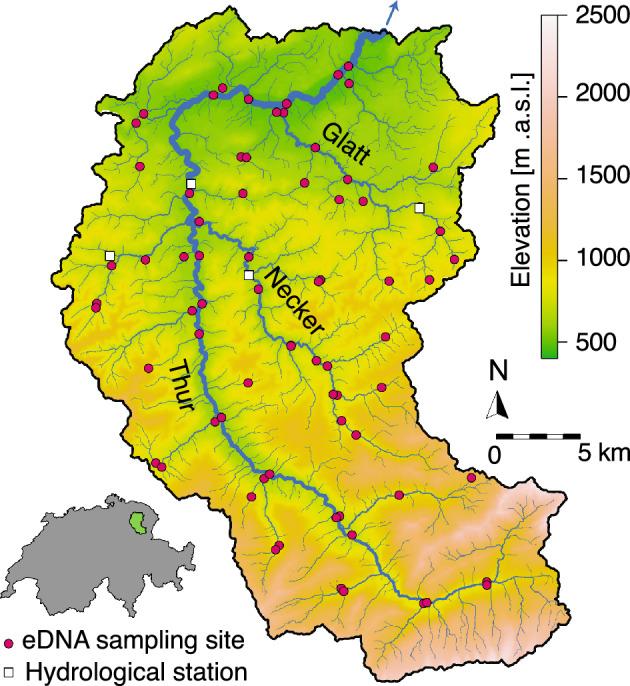
Map of the Thur catchment displaying the locations of eDNA sampling sites (red dots) and hydrological stations (white squares). The three main tributaries (rivers Thur, Glatt and Necker) are also identified. The blue arrow identifies direction of water flow from the river outlet. The map was generated via the *rivnet* R-package^46^.

Here, we illustrate the potential of eDITH in detecting such temporal and taxonomically broad trends of riverine biodiversity. To do so, we built on a previous application^43^ of eDITH to a Swiss catchment (the Thur, Fig. 1). While ref.^43^ has looked at selected macroinvertebrates at a single season, we now extend this by using a novel eDNA metabarcoding dataset^45^ covering three broader taxonomic groups relevant to freshwater communities (fish, invertebrates and bacteria), and three seasons (spring, summer and autumn) collected at 73 sites across the Thur catchment. The eDITH model enabled predicting patterns of *α*- and *β*-diversity, and changes thereof with respect to season and major taxonomic groups, at an unprecedented space-filling, highly resolved spatial scale (1908 river reaches of length $\sim$500 m). We then compared the so-obtained (hereafter termed “predicted”) diversity patterns with those derived by directly analyzing the “raw” eDNA data. This provided insights on the added value of using eDITH to correctly interpret eDNA data in rivers, thus highlighting a way forward for enhanced freshwater biodiversity monitoring and conservation.

**Table 1:** Summary of the effect of drainage area (i.e. position within network) on predicted and raw *α*- and *β*-diversity patterns. $\nearrow$: increasing in the downstream direction; $\searrow$: decreasing in the downstream direction; $\rightarrow$: invariant relationship; $^*$: significant relationship; $^{NS}$: non-significant relationship; −: not assessed. Approaches to detect significance are detailed in the “Methods”.

		*α* diversity			Spatial *β* diversity			Temporal ${\beta}$ diversity	
		spring	summer	autumn	spring	summer	autumn	spr.-sum.	spr.-aut.
Predicted	Fish	$\nearrow ^*$	$\nearrow ^*$	$\nearrow ^*$	$\rightarrow$	$\searrow ^*$	$\nearrow ^*$	$\nearrow ^*$	$\nearrow ^*$
	Invert.	$\searrow ^*$	$\searrow ^*$	$\nearrow ^{NS}$	$\rightarrow$	$\rightarrow$	$\searrow ^*$	$\nearrow ^*$	$\nearrow ^*$
	Bacteria	$\nearrow ^{NS}$	$\searrow ^*$	$\nearrow ^{NS}$	$\searrow ^*$	$\searrow ^*$	$\searrow ^*$	$\nearrow ^*$	$\nearrow ^{NS}$
Raw	Fish	$\nearrow ^*$	$\nearrow ^{NS}$	$\nearrow ^*$	-	-	-	$\nearrow ^{NS}$	$\searrow ^{NS}$
	Invert.	$\nearrow ^{NS}$	$\searrow ^{NS}$	$\searrow ^{NS}$	-	-	-	$\nearrow ^{NS}$	$\searrow ^{NS}$
	Bacteria	$\nearrow ^{NS}$	$\searrow ^*$	$\searrow ^{NS}$	-	-	-	$\nearrow ^*$	$\searrow ^{NS}$

**Figure 2:**
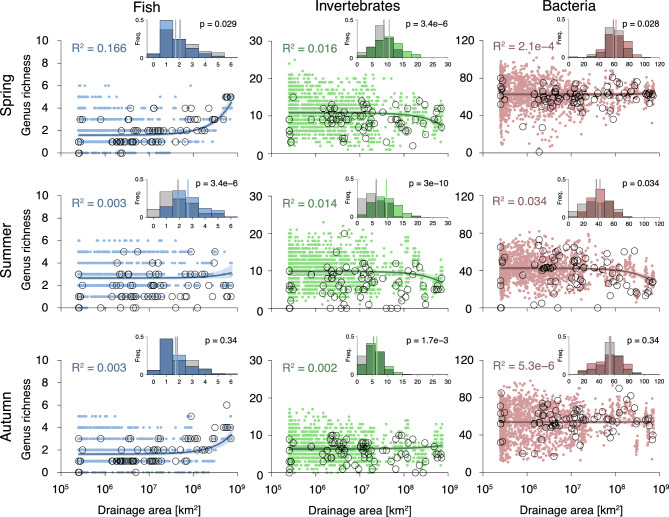
Patterns of *α*-diversity as a function of drainage area. Colored, closed dots represent eDITH model results; for comparison, values inferred from eDNA data are displayed with black, open dots. Colored solid lines represent linear model fits on predicted genus richness (corresponding $R^2$ values are reported on the top-left corner). Shaded areas represent 95% confidence intervals on linear model fit (obtained via a bootstrapping technique detailed in the “Methods”). Note that linear models were fitted on natural values on drainage area, hence the trend lines are exponential in these semi-logarithmic plots. Inset: frequency distribution of genus richness values as predicted by eDITH (colored bars) vs. inferred from eDNA data (grey bars). Solid vertical lines display means of the distributions; *p*-values are obtained from *t*-tests.

**Figure 3:**
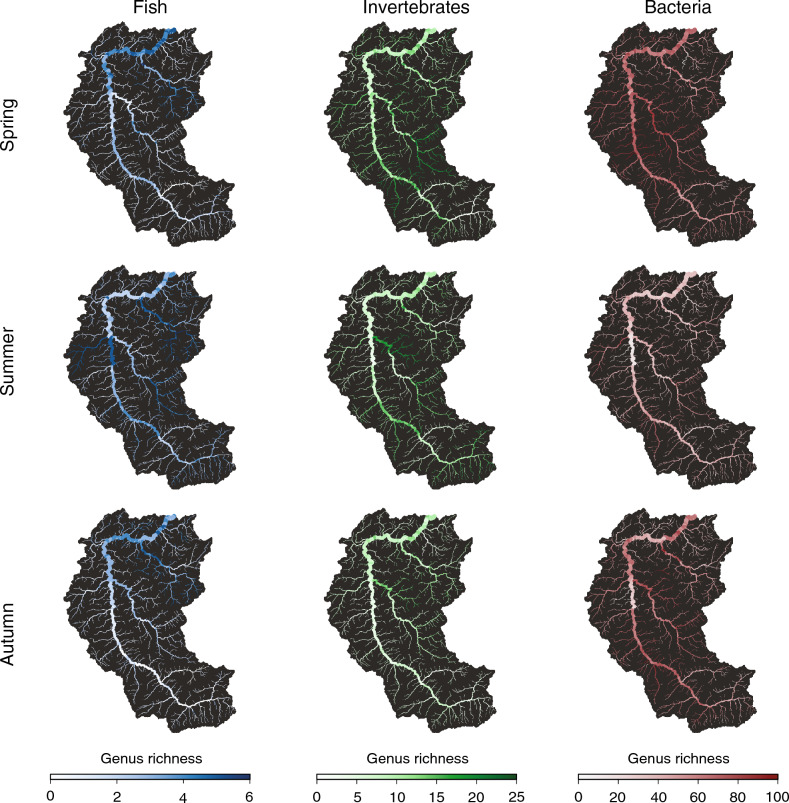
Spatial patterns of predicted *α*-diversity (expressed in terms of genus richness) for the different taxonomic groups and seasons. Displayed values correspond to colored, closed dots in Fig. 2. Maps were generated via the *rivnet* R-package^46^.

## Results

### *α*-diversity patterns

Overall, predicted patterns of *α*-diversity across the different taxonomic groups and seasons show weak, and often non-significant relationships with drainage area (Table 1, Fig. 2). Indeed, the proportion of variance in predicted *α*-diversity explained by drainage area is in all cases lower than 4%, with the exception of fish in spring, where drainage area explains 16.6% of the variance in predicted *α*-diversity (Fig. 2). Conversely, patterns of predicted *α*-diversity appear to be driven by clusters of neighbouring reaches: for instance, the main stem of the Thur right upstream of the confluence with the Necker is predicted to be particularly depleted in bacterial taxa in autumn, but rich in fish in summer (Fig. 3). This is reflected by the role of environmental covariates in explaining the spatial patterns of taxa (see Supplementary Fig. S1 for a summary of significant covariates depending on taxonomic groups and seasons).

In general, predicted genus richness is higher than the raw one (Fig. 2, insets): across seasons and taxonomic groups, the mean predicted *α*-diversity is significantly higher ($p<0.05$ on a *t*-test) than the raw one in six cases out of nine; in two cases (fish and bacteria in autumn) the difference between means is not significant, and only for fish in spring is the raw mean *α*-diversity significantly higher than the predicted one. Moreover, for fish and invertebrates and irrespective of the season, predicted *α*-diversity is higher than the raw one for low drainage areas (Fig. 2). The effect of drainage area on raw *α*-diversity does not generally match the trends observed with respect to predicted *α*-diversity (Table 1): in particular, raw *α*-diversity is not significantly correlated with drainage area for fish in spring and invertebrates in spring and summer, as opposed to predicted *α*-diversity.

Predicted fish *α*-diversity is significantly correlated with drainage area in all seasons (Table 1, Fig. 2). While fish genus richness in spring and autumn is mostly concentrated in the downstream reaches of the watershed, richness in summer is highest in small tributaries at intermediate distance from the river outlet (Fig. 3). For invertebrates, the trend of predicted *α*-diversity decreases significantly in the downstream direction in spring and summer, while it increases non-significantly in autumn (Table 1, Fig. 2). Clusters of high invertebrate *α*-diversity are predicted in the mid-Thur and Necker reaches, which is in qualitative agreement with the patterns found by ref.^43^ for the orders Ephemeroptera, Plecoptera and Trichoptera in late June (note that, in the dataset here analyzed, 29 invertebrate genera out of 80 belong to one of these three orders). Invertebrate genus richness in autumn tends to be lower as compared to spring and summer (Figs. 2, 3). This finding is reflected by the sensibly lower number of reads observed in autumn for invertebrates as compared to the other seasons, although the total number of invertebrate genera detected in autumn (56) is comparable to that of spring (62) and summer (57) (see Supplementary Fig. S2). Predicted bacteria *α*-diversity shows a flat distribution across the catchment in all seasons, with a significant decrease in the downstream direction observed only in summer, while the effect of drainage area is not significant in spring and autumn. Summer values of predicted bacterial *α*-diversity are considerably lower with respect to spring and autumn (Figs. 2, 3).

Across all taxonomic groups and seasons, the percentage of moraines upstream (G-MO) is one of the most important predictors of the spatial distribution of taxa (Supplementary Fig. S1), with either positive or negative effects depending on the specific group and season: for bacteria, remarkably, such covariate has a strong positive effect in spring and autumn, but a strong negative effect in summer. Moraines are mostly located in the lowland areas of the catchment (Supplementary Fig. S3), hence such result could be indicative of a spurious correlation (and was already observed for another application of the eDITH model in a Swiss prealpine river^41^). Covariates identifying river reaches belonging to the Necker tributary (NE1, NE2, NE3 - Supplementary Fig. S3) have a strong positive effect for invertebrates across all seasons, indicative of high richness of this taxonomic group in this area.

Patterns of predicted *α*-diversity for any taxonomic group are positively correlated across seasons, with spring-autumn correlations being higher than spring-summer correlations for all taxonomic groups (Supplementary Fig. S4). Moreover, invertebrate *α*-diversity is strongly correlated with bacteria *α*-diversity in all seasons, suggesting a link between these contiguous trophic levels; fish *α*-diversity also shows positive correlations with invertebrate *α*-diversity in all seasons, although the effect is in this case less strong (Supplementary Fig. S5).

**Figure 4:**
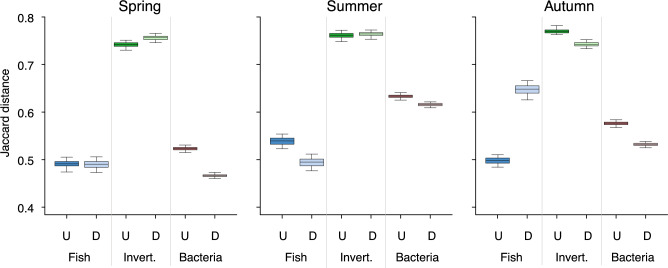
Effect of drainage area on predicted spatial *β*-diversity of fish, invertebrates (“Invert.”) and bacteria, respectively. Each boxplot contains 100 values, representing the mean pairwise Jaccard distance for one of the 100 replicated choices of pairs within a group of reaches (“U”: upstream; “D”: downstream - see “Methods”). Boxes’ extent corresponds to interquantile range; whiskers’ extent to 2.5th-97.5th percentile range.

**Figure 5:**
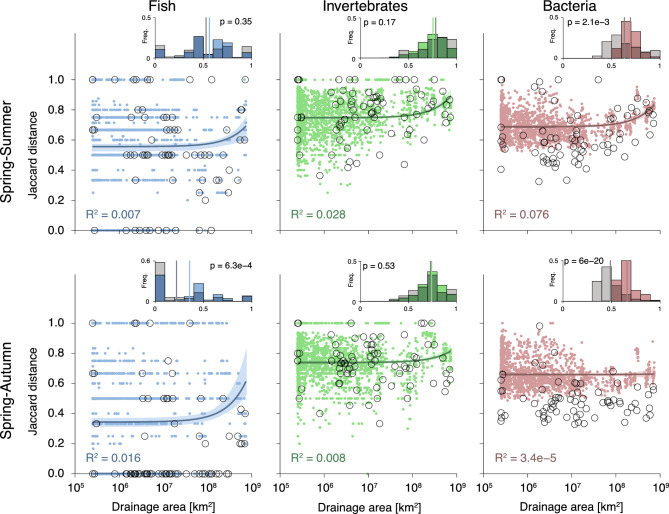
Patterns of predicted temporal ${\beta}$-diversity as a function of drainage area. Figure construction is as in Fig. 2.

**Figure 6:**
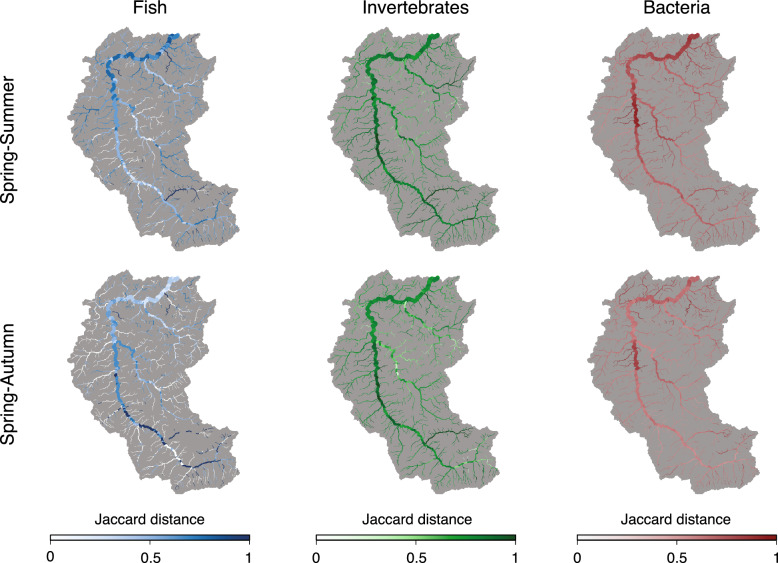
Spatial patterns of predicted temporal ${\beta}$-diversity (expressed via the Jaccard distance). Maps were generated via the *rivnet* R-package^46^.

### ${\beta}$-diversity patterns

The effect of drainage area on predicted spatial *β*-diversity patterns depends greatly on the taxonomic group and the season (Table 1, Fig. 4). For fish, spatial ${\beta}$-diversity decreases in the downstream direction in summer, while the pattern is reversed in autumn, and no effect of drainage area is observed in spring; for invertebrates, there is no significant trend of drainage area on spatial *β*-diversity in spring and summer, while a decreasing trend is observed in autumn; finally, spatial ${\beta}$-diversity of bacteria decreases significantly with drainage area in all seasons. Importantly, across all seasons, values of the Jaccard distance for invertebrates are much larger than those for fish and bacteria. Analysis of the relative contribution of nestedness and turnover components to the total Jaccard distance shows the predominant role of turnover in ${\beta}$-diversity for invertebrates and bacteria across all seasons, while a larger role of nestedness is observed for fish (Supplementary Table S1).

Patterns of predicted temporal ${\beta}$-diversity are significantly and positively related to drainage area for all taxonomic groups and seasons, with the exception of bacteria in the spring-autumn comparison, where the positive trend is not significant (Table 1, Fig. 5). However, the proportion of variance in predicted Jaccard distance explained by drainage area is low (i.e. consistently lower than 8%). Interestingly, for all taxonomic groups, higher values of predicted temporal ${\beta}$-diversity are observed in the spring-summer, than in the spring-autumn comparison (Supplementary Table S2). This reflects the fact that patterns of predicted spring *α*-diversity are better correlated to patterns of autumn, than summer *α*-diversity (Supplementary Fig. S4). Moreover, values of predicted temporal *β*-diversity for invertebrates are higher than those for fish and bacteria, thus showing that predicted patterns of invertebrate communities (or their detectability, see Discussion) are much more diverse in both spatial and temporal dimensions than other taxonomic groups. For fish, larger values of predicted Jaccard distance in the spring-summer comparison are observed at the downstream reaches, while the highest temporal ${\beta}$-diversity is found at the mid-to-upper Thur reaches (Fig. 6). Regarding invertebrates, the predicted spatial trends of temporal ${\beta}$-diversity tend to be more constant across seasons, with larger values observed along the main Thur stem, and more stable communities in the Necker and Glatt tributaries (Fig. 6). Predicted bacteria temporal ${\beta}$-diversity shows a less pronounced spatial variability, with all values being larger than 0.4 in all seasons (Fig. 5). As it was the case for spatial ${\beta}$-diversity, predicted temporal ${\beta}$-diversity of fish is mostly driven by nestedness, while turnover plays a major role in the temporal ${\beta}$-diversity of invertebrates and bacteria (Supplementary Table S2). The predominance of the nestedness component in fish ${\beta}$-diversity reflects their higher propensity to dispersal compared to other taxonomic groups.

Finally, bacteria temporal ${\beta}$-diversity evaluated on the raw eDNA data is considerably lower with respect to the predicted one (Fig. 5, insets: $p<0.05$ for both spring-summer and spring-autumn comparisons); such differences are due to a larger weight of the turnover component for the predicted Jaccard distances (Supplementary Table S2). As for fish and invertebrates, differences between the two approaches are less marked, with only predicted temporal ${\beta}$-diversity in the spring-autumn comparison being significantly higher than the raw one; however, raw temporal ${\beta}$-diversity tends to assume more extreme (both low and high) values than the predicted one. Notably, unlike the predicted temporal ${\beta}$-diversity, raw temporal ${\beta}$-diversity is in most cases not significantly correlated with drainage area, the only exception being bacteria in the spring-autumn comparison (Table 1).

## Discussion

The combined use of the eDITH model and a spatially and temporally replicated multimarker eDNA dataset allowed the assessment of patterns of *α*- and *β*-diversity across a river catchment with approximately a 30-fold increased resolution compared to the initial sampling sites. Specifically, using data from 73 sampling sites across the whole catchment, diversity patterns across all taxonomic groups analyzed in this study were extrapolated at a genus level to cover a total of 1908 river reaches (of mean length $\sim$500 m) along the complete river network, giving unprecedented spatially covered information on fish, invertebrates and bacteria. The combined and integrated study of major organismal groups, including microbes, invertebrates and vertebrates, in a common methodological approach and framework is largely unprecedented and enables comparison of biodiversity patterns of hitherto mostly individually studied organisms. Comparison of these *α*- and temporal *β*-diversity patterns obtained via the eDITH model vs. the raw eDNA data revealed substantial differences between these two approaches for several taxonomic groups and seasons, both in terms of means of the distribution of values and of direction and significance of the trend in the downstream direction. As for spatial *β*-diversity patterns, a proper comparison with the raw data could not even be done due to the (inevitably) limited number of eDNA sampling sites available (see “Methods”). It is widely acknowledged that performing richness and diversity studies on raw eDNA data can be problematic due to the possibly large number of false negatives arising from the multiple steps of the metabarcoding procedure^47^. With eDITH, this aspect is accounted for via the assumption of geometric distribution of observed read number values for a given site and taxon. This allows detecting components of richness and diversity that would be otherwise overseen. Indeed, we found genus richness values estimated via eDITH to be often higher than those assessed from the raw data (Fig. 2).

Importantly, we found patterns of predicted *α*- and *β*-diversity to be strongly dependent both on the taxonomic group and season, which is in contrast with the predictions of the river continuum concept^9^ and the network positioning hypothesis^18,19^ of seasonally invariant and increasing *α*-diversity and decreasing *β*-diversity in the downstream direction, respectively. The only taxonomic groups for which patterns were found to be in accordance with these predictions are fish for *α*-diversity and bacteria for *β*-diversity. The lack of support for the network position hypothesis shown by our results on spatial *β*-diversity is actually in line with the high unexplained variance observed in many field studies (e.g., refs.^21,24^). Overall, however, drainage area was found to be a poor (and often even non-significant) predictor of patterns of both diversity types. Indeed, occurrence of genera resulted to be correlated to a variety of environmental covariates, with considerable variation in importance and direction of the effect across seasons and taxonomic groups (see Supplementary Fig. S1). On the same dataset, ref.^45^ assessed effect of drainage area and season on genus richness via mixed models (applied to the raw eDNA data), finding a positive, significant effect of drainage area for fish, and a significant effect for bacteria whose direction depended on the season. Our modelling approach came to a similar conclusion with respect to these groups, but additional negative, significant effects of drainage area for invertebrates in spring and summer were observed (Table 1). Notably, a decreasing trend of bacterial richness in the downstream direction had already been observed in some river systems (e.g. ref.^15,16^) and linked to the increased ratio of benthic area to water volume in headwaters, which is likely to promote transport of terrestrial microorganisms into stream habitats.

The prominent role of environmental covariates such as upstream moraine coverage also offers a plausible explanation for the poor predictive power of drainage area with respect to the diversity patterns. Indeed, areas of the catchment that are rich in moraines mostly coincide with sites close to the river outlet. An upstream-to-downstream gradient based on distance to the outlet is thus likely to confound the expected patterns of increasing richness with drainage area. Indeed, small tributaries close to the outlet might show higher *α*-diversity than those far from it, possibly due to both mass effects and increased nutrient availability in lowland, agriculture-dominated areas. Reaches with low drainage area are found all across the watershed, and they are characterized by a variety of abiotic conditions (elevation, slope, temperature, substrate etc.), which likely enhances the variability of *α*-diversity values therein.

As for temporal *β*-diversity, to our knowledge, no general theories for spatial patterns thereof in river networks have been formulated. However, some recent studies^48^ hinted that temporal fluctuations in population abundances might be more attenuated in downstream than in upstream, flow-unconnected sites. If translated to community dynamics, this would be suggestive of a lower temporal *β* -diversity in the downstream direction. However, our findings do not support the hypothesis of temporal ${\beta}$-diversity decreasing downstream, and rather point towards the opposite conclusion of a downstream-increasing trend. Nevertheless, the fraction of variance in temporal ${\beta}$ -diversity explained by drainage area is quite low, and hence the support for this reversed trend is also limited. Further studies would be required to assess to which extent these results are specific to the examined study area and taxonomic groups, and/or affected by our sampling methodologies and model’s assumptions.

Predicted patterns of invertebrates were found to be highly variant in both space and time, as spatial and temporal ${\beta}$-diversity values were the highest with respect to other taxonomic groups (Figs. 4, 5, Supplementary Tables S1, S2). Indeed, most invertebrate genera were predicted to be located only in limited parts of the catchment and showed a marked temporal variability. Only 2.5% of the invertebrate genera (i.e. 2 out of 80, *Baetis* and *Eiseniella*) were predicted to be present in at least 25% of the same reaches across the three seasons (Supplementary Fig. S6). In contrast, bacteria communities showed lower variability in both space and time, with a core group of genera that were found to be present in large portions of the catchment irrespective of the season: 9.6% of the detected genera (i.e. 27 out of 282) occupied at least 25% of the same reaches in all seasons (Supplementary Fig. S6). A plausible explanation for the limited variability in bacteria patterns is the fact that bacteria genera generally consist of a higher number of species than is the case for the other taxonomic groups, hence possible turnover at the species level is here hidden. Higher variability of invertebrate patterns as compared to bacteria is also supported by the fact that predicted invertebrate richness in spring explains less variation in richness in the other seasons as compared to bacteria (and partially to fish, see Supplementary Fig. S4). Fish were found to be the most stable taxonomic group, with temporal ${\beta}$-diversity values much lower than those for invertebrates and bacteria (Fig. 5, Supplementary Table S2), and spatial values lower than those for invertebrates and comparable to those for bacteria (Fig. 4, Supplementary Table S1). This result is likely influenced by the limited number (12) of fish genera detected, and the ubiquity of the genus *Salmo*, which was predicted to be present in $>80\%$ of the same reaches across the three seasons (Supplementary Figs. S6,  S7).

Overall, we see two main mutually non-exclusive explanations for these patterns and their limited overlap with previous models and findings^9,18^. First, past predictions of riverine diversity patterns were often based on a small number of sites, often situated in a linear line-up along the main river stem. Thereby, these studies did not consider contributions of small-scale spatial dynamics from the dendritic river network, nor temporally fluctuating dynamics in organisms’ abundance and occurrence. Such time-invariant assumptions may be adequate for some groups, yet are likely not realistic for systems with a pronounced seasonality, including alternation between high and low flows or even desiccation, resulting in complex population dynamics. Second, our approach assumes an even detectability across space for the taxa considered. Likely, this strong assumption is at least to some degree violated, as eDNA-based data are known to be highly affected by stochasticity^49–51^ or may not be totally replicable, especially with generic primers as used here. Thus, some of the observed patterns may also reflect heterogeneity in the sampling procedure itself. While we cannot separate these processes, we are confident that the latter (uneven detectability) does not play a dominant role. Note also that possible temporal differences in detectability have been accounted for by the modeling procedure, since taxon distribution patterns were obtained separately for each season, and by using time-specific values for the hydrological variables. Therefore, despite eDNA shedding rates being dependent on environmental factors such as water temperature and metabolic activity^52,53^ and hence arguably on season, these aspects do not affect our predictions.

In particular, seasonal differences in diversity patterns could be related to biological (when taxa actually change their abundance and/or spatial distribution across seasons) or methodological (when the likelihood to detect taxa changes across seasons) aspects. In the latter case, absence data may not be indicative of a true absence, but may be interpreted as an “ecological absence” (i.e. not ecologically relevant at that time point due to low density^45^). In our results, several observed patterns have a plausible biological explanation. First, the higher *α*-diversity of fish in low size reaches in summer as compared to the other seasons (Fig. 3) is possibly due to the migrating behaviour of several fish taxa during the spawning season, which, for widely found species in European temperate rivers, such as the gudgeon (*Gobio gobio*) and the common minnow (*Phoxinus csikii*), happens in late spring and early summer (see the predicted presence/absence maps for genera *Gobio* and *Phoxinus* in Supplementary Fig. S7). Second, the lower *α*-diversity of invertebrates in autumn (Fig. 3) is likely related to the fact that many aquatic insects have already completed the aquatic part of their life cycle in autumn. Instead, the observed lower bacteria richness in summer compared to spring and autumn could have a methodological explanation: indeed, the total number of reads observed for bacteria in summer is intermediate with respect to those for spring and autumn, resulting however in a lower number of detected genera (198) with respect to spring (220) and autumn (214) (Supplementary Fig. S2). This apparent mismatch between read number count and number of detected genera could be explained by the proliferation of some bacterial taxa in summer following temperature increase, which could mask the DNA from rarer taxa in the sequencing procedure, such that most (or all) amplification is biased towards the dominant taxa. Such patterns, similarly to primer bias, are well known in metabarcoding studies^51^, and we acknowledge that the relatively low volume of water sampled may not have resulted in sufficiently saturated species accumulation curves.

The use of the eDITH model allowed an enhancement of our capability of interpreting eDNA data, enabling the extraction of patterns of *α*- and *β*-diversity at a much higher spatial resolution than what could be achieved by analyzing the eDNA data alone. Indeed, the spatial resolution at which model predictions are produced can be tuned freely, and this choice does not add any complexity to the model fitting procedure, as long as eDNA production rates $p_i$ are expressed as a function of environmental covariates. In this application, we imposed a maximum reach length of 1 km, which resulted in a total of 1908 reaches, more than double than the number of reaches (760) used in a previous application in the same catchment^43^. However, it is important to note that a finer discretization of the river network would result in predicted richness values at the different reaches that would be more interdependent, which would require adequate tools (such as the ad-hoc statistical tests performed here) to analyze the resulting patterns. Another caveat in this respect, pointing at an opposite direction, is that too fine of a discretization might result in unrealistic small-scale differences in taxon patterns (e.g., presence-absence-presence predicted at a sequence of short, flow-connected reaches); a potential solution is offered by roughness-minimizing approaches borrowed by fluvial geochemistry in the analogous problem of predicting geochemical maps in catchments based on downstream water samples^54^.

We acknowledge that the herein assessed riverine biodiversity patterns do not cover sampling variability at the site level; it is indeed known that read number values vary substantially as a result of stochasticity in the sequencing process^49,50,55^, and thus our results contain some level of stochastic variation that we cannot control for. Higher robustness of eDITH predictions would be possible by incorporating true sampling replicates of the eDNA water samples, upon which the model fitting procedure is applied. However, we note that, in our analysis, eDITH’s predictions for a given genus and time point are based on the ensemble of 73 spatially distributed samples, and hence they take into account (at least partially) the stochasticity of the metabarcoding data, even in the absence of true sample replicates.

The accuracy of eDITH-based predictions was extensively and successfully tested in ref.^43^, where the model was applied to a similar dataset from the same river system, covering a smaller subset of macroinvertebrates (may-, stone- and caddis-flies) at a single time point. That study assessed the accuracy both in terms of overlap between eDITH predictions and direct observation of organisms (i.e. an independent dataset; finding an average percentage of true predictions across taxa of 82.4%), and as robustness to changes in the number of sampling points used to fit the model. With respect to this latter point, we also extensively verified the suitability of our sampling design in a theoretical study^44^. Moreover, we note that, for specific genera for which we have detailed biological and natural history knowledge (in particular, as commented above, fish genera such as *Phoxinus*, *Gobio* and *Salmo*), we found predicted spatial distributions to be in strong agreement with expected seasonality of their migration patterns (Supplementary Fig. S7).

Validation of eDITH predictions with abundance estimates from direct observation of organisms would in principle be feasible for fish (e.g., via electrofishing) and invertebrates (via kicknet sampling), at least for one time point (see also ref.^32^), although invasive and cumbersome (see Introduction). Conversely, validation of bacterial patterns is inherently depending on metabarcoding approaches, possibly from samples extracted from different substrates (e.g., biofilm, although free floating bacteria communities can arguably not fully overlap with communities growing in benthic biofilm). In this respect, a possible improvement of the eDITH model would consist in the merging of eDNA and direct organismal observation data via joint-likelihood data integration methods^56,57^. Another conceivable expansion of the current analysis regards the use of taxon richness predictions provided by eDITH to foster food web analysis at an unprecedented spatial resolution (see ref.^45^ for assessment of food web characteristic and functional diversity based on the raw eDNA data). This analysis seems particularly promising in the case study at hand, given the clear link that we observed between richness patterns of contiguous trophic level (Supplementary Fig. S5).

The eDITH approach is not restricted to a specific catchment, as its application, besides spatially replicated eDNA data, only requires widely available geographic (digital elevation models and possibly land cover maps) and hydrological information (an estimate of water discharge at a single point in space is sufficient). Moreover, eDITH is also not restricted to the type of taxa analyzed, as it does not rely on specific hypotheses on eDNA dynamics in water or sequencing aspects. This is demonstrated by our study, which analyzed about 400 genera from three very different, major taxonomic groups. Such combined assessment of biodiversity estimates by one common method is virtually impossible if not by using eDNA. Due to hydrological transport, eDNA measures of biodiversity collected from stream water are lumped across a potentially wide upstream area: the use of eDITH-based predictions of biodiversity hence allows a complete re-interpretation of the raw eDNA data. Therefore, the eDITH approach is generally applicable to any taxonomic group in riverine ecosystems and any temporal window, providing biodiversity predictions that can be used in the analysis of ecosystem processes, as well as to implement targeted (both spatially and temporally) conservation interventions. In our case study, predicted patterns of *α*- and ${\beta}$-diversity across taxonomic groups and seasons were not generally amenable to a simple increasing or decreasing trend in the downstream direction, but rather to a complex interplay of environmental variables, abiotic and biotic factors, highlighting the need for differentiated conservation approaches in riverine systems. A thorough assessment of biological communities in rivers cannot forego the integration of these aspects, and the eDITH model can be an adequate tool for such integrated analyses.

## Methods

### Study area

The Thur (Fig. 1) is a pre-alpine river located in northeastern Switzerland, draining an area of 740 km^2^, for which extensive data on hydrology and biodiversity are available^43,45,58,59^. Here, eDNA samples were collected at 73 sites in three different seasons in 2018: spring (17th-24th May), summer (20th Aug-5th Sep), and autumn (2nd-8th Oct). In summer, 4 sites were not sampled because their respective reaches were temporarily dry. Hydrological data on the catchment were available at four gauging stations (see Fig. 1) operated by the Swiss Federal Office for the Environment, while landscape data on elevation, land cover types and geology were provided by the Swiss Federal Office for Topography (see ref.^43^ for details).

The river network was extracted by using a TauDEM implementation of the D8 method^60^ on a 25-m digital elevation model of the region. A threshold drainage area of 0.25 km$^2$ was used to identify the sources of the river network, resulting in a total river length of 1034 km. Such threshold area value was chosen as the maximum one such that all 73 sampling sites effectively belonged to the resulting river network. The river was partitioned into reaches, i.e. river segments not interrupted by confluences, which were considered as habitat ranges of local communities. We imposed a maximum reach length of 1 km by following ref.^61^, which resulted in a total of 1908 reaches, with mean length 542 m.

### eDNA data collection and sequencing

Environmental DNA samples were collected and filtered on site using disposable 50 mL syringes and 0.22 $\mu$m sterivex filters (Merck Millipore, Merck KgaA, Darmstadt, Germany). At each site 1 L of water was filtered, which is a sufficient water volume for eDNA sampling in rivers of the size of the Thur^62^. Samples were extracted in clean lab facilities using the DNeasy PowerWater Sterivex Kit (Qiagen, Hilden, Germany) following the manufacturer’s protocol. Three libraries were constructed for the following markers: 12S targeting fish (forward primer sequence: 5’- TACTGGGATTAGATACCCC-3’ and reverse primer sequence: 5’- CTAGAACAGGCTCCTCTAG-3’)^63^ targeting fish, COI targeting macroinvertebrates (forward primer sequence: 5’- GGWACWGGWTGAACWGTWTAYCCYCC-3’ and reverse primer sequence: 5’- TAIACYTCIGGRTGICCRAARAAYCA-3’)^64,65^ and 16S targeting bacteria diversity (forward primer sequence: 5’- CCTACGGGDGGCWGCA-3’ and reverse primer sequence: 5’- GACTACHVGGGTMTCTAATC-3’)^66^, using a two-step (12S and COI) and three-step (16S) library preparation method. Cleaned amplicons were indexed using unique combinations of the Illumina Nextera XT Index Kit (Illumina, Inc., San Diego, CA, USA) A, C and D in the last PCR, following the manufacturer’s protocol. Paired-end sequencing was performed on an Illumina MiSeq (Illumina, Inc. San Diego, CA, USA) at the Genetic Diversity Centre at ETH, Zurich (see ref.^45^ for full details of each library preparation). After each of the libraries were sequenced, the data of each library was demultiplexed and reads were quality checked. Raw reads were end-trimmed, merged and quality filtered, additional reads were clustered at 99% identity to obtain error corrected and chimera-filtered sequence variants ZOTUs (Zero-radius Operational Taxonomic Units). The final ZOTUs were then clustered using a 97% similarity approach and taxonomic assignment with a 0.85 confidence threshold. Taxonomic assignment was carried out with a 0.85 confidence threshold and was performed using SINTAX in the usearch software v11.0.66764^67^. For each of the datasets we used the following publicly available reference databases: NCBI BLAST (v200416) for 12S; a custom reference database (including MIDORI un-trimmed (V20180221)) for COI; SILVA (V128) for 16S. For full details of the bioinformatic parameters used see ref.^45^. Prior to data analysis a contamination threshold was applied to each sample, whereby 0.1% of reads in each sample was removed from each genus present in that sample. This method is used to reduced possible contamination and tag-jumping during library preparation and sequencing and has been shown as an effective contamination thresholds^59,68,69^. All genera with a non-aquatic life stage were also removed from the dataset and the data was merged at genus level.

We decided to focus on genus as our target taxonomic level, as this level is the best compromise with respect both to coverage in reference databases and ecological relevance (species within a genus often share similar traits, thus are functionally similar). Importantly, for fish, most expected species belong to different genera within a given biogeographical region (as documented for Swiss fresh waters in ref.^70^), thus genus-level identification often also allows species-level interpretation, while for invertebrates the database coverage was best for genus level. Note that, in principle, the same analyses could have been operated on taxonomically unspecified clusters such as molecular OTUs (MOTUs), but this would have hindered interpretation of the results from a conservation perspective, where the relevant units are described taxa (species or genera).

Overall, we detected 12 fish, 80 invertebrate and 282 bacterial genera (see Supplementary Table S3 for the complete list of genera). The three fish genera *Barbus*, *Gobio* and *Phoxinus* were detected with both the 12S and COI barcode regions. To avoid duplicated coverage, we removed from the database the read numbers corresponding to these three genera from the COI library, as the corresponding read numbers in the 12S library were higher and the latter marker region was used to target fish. Supplementary Fig. S2 displays the total number of reads observed and the total number of genera detected for each barcode region, taxonomic group and season, pooled over all sampling sites.

### eDITH model

The eDITH model implementation essentially follows ref.^43^, and is here summarized for the specific study setting. For each genus the expected eDNA concentration $C_j$ at a sampling site *j* of the network reads:

1 $$\begin{aligned} C_j = \frac{1}{Q_j} \sum _{i \in \gamma (j)} p_i A_{S,i} \exp {\left( -\frac{L_{ij}}{\overline{v_{ij}}\tau }\right) }\,, \end{aligned}$$

where $Q_j$ is the water discharge at reach *j* (i.e. the reach where sampling site *j* is located), $\gamma (j)$ identifies the set of reaches upstream of *j* (with *j* included), $p_i$ the eDNA production rate at reach *i*, $A_{S,i}$ the source area of reach *i* (namely its open water surface), $L_{ij}$ the along-stream path from *i* to *j*, $\overline{v_{ij}}$ the average water velocity along such path, $\tau$ a characteristic decay time. eDNA production rates are expressed via a Poisson generalized linear model as $p_i=p_0 \exp {\left( \varvec{{\beta} }^T \textbf{X}(i) \right) }$, where $\textbf{X}(i)$ is a vector of environmental covariates, $\varvec{{\beta} }$ a vector of covariate effects and $p_0$ a baseline production rate. We utilized 35 covariates, representing morphological, land cover, geological and geographical characteristics of the catchment. These covariates correspond exactly to those used in ref.^43^ (Supplementary Fig. S2). Observed read data from each genus at a given site *j* and a given season were assumed to follow a geometric distribution, with mean proportional to $C_j$. Following ref.^43^, reach width was evaluated via aerial images in correspondence to the four hydrological stations, and a power-law relationship with drainage area was derived. Width values were then extrapolated to all 1908 reaches via the so-obtained power-law relationship. The same procedure was performed for the three different seasons for discharge and water depth, whose data values were taken as the averages of the mean daily measured values at the hydrological stations during the respective sampling periods. A power-law relationship on drainage area was then fitted separately for each hydrological variable (i.e. discharge, water depth) and season, and then extrapolated to the whole catchment. Finally, we calculated water velocity values at all reaches for all seasons under the hypothesis of rectangular river cross-sections (i.e. $v=Q/(wd)$, where *v* is velocity, *Q* is discharge, *w* is width and *d* is depth).

The posterior distributions of the 37 unknown parameters (i.e. vector $\varvec{{\beta} }$ containing effect sizes for 35 covariates, decay time $\tau$ and baseline production rate $p_0$) were inferred independently for each season and genus, by using the DREAM$_{ZS}$^71^ algorithm, implemented via the *BayesianTools* R-package^72^. Three independent Markov chains were run, with a total chain length of $3\cdot 10^6$ (plus a burn-in length of $5\cdot 10^5$). A normal prior distribution with null mean and standard deviation of 3 was adopted for all $\varvec{{\beta} }$ components; $p_0$ had a uniform prior bound between 0 and 1; a log-normal prior for $\tau$ was chosen, with a median of 5 h and a mode of 4 h. The so-obtained maximum a posteriori parameter estimates were used to produce maps of relative species density (i.e. $p_i$). These were subsequently translated into detection probability maps by evaluating the expected read number that would be observed at a reach if the reach were disconnected from the river network, and by assessing the probability that the measured read number therein would be larger than 0 according to the assumption of geometric distribution of read numbers (see ref.^43^ for details). Finally, presence/absence maps for each genus were derived by imposing a threshold of 0.5 on detection probability.

### Evaluation of *α*- and *β*-diversity patterns

For each taxonomic group and season, the number of genera predicted by the eDITH model to be present in each of the 1908 reaches was taken as a measure of -diversity. We then performed a linear regression to assess the effect of drainage area on genus richness. Given that values of genus richness at the different reaches are in principle not independent (i.e. due to the spatial structure of the covariates used to predict the taxon patterns, predicted *α*-diversity values for nearby reaches tend to be correlated), we refrained from performing classic statistical tests on the slope of the linear regression. Instead, we assessed the significance of the effect of drainage area via a bootstrapping approach: we subsampled 500 out of 1908 reaches in a quasi-random fashion (i.e. by splitting reaches into 10 bins according to the drainage area deciles, and randomly sampling (with replacement) 50 reaches within each bin), and linearly regressed genus richness on drainage area for the subsampled reaches. We repeated this procedure 100 times, and considered a positive (respectively negative) significant effect of drainage area if, in at least 95 out of 100 cases, the fitted slope of the linear regression was positive (respectively negative). The 2.5th-97.5th percentile range of the so-obtained 100 linear regression lines was used as confidence interval of the linear model fit. Moreover, we also computed genus richness directly from the eDNA data at the 73 sampling sites across seasons and taxonomic groups.

We assessed spatial patterns of ${\beta}$-diversity with respect to each of the three major taxonomic groups by comparing pairwise Jaccard distances across the 1908 river reaches within two location groups. Specifically, reaches were assigned to the “upstream” group, if their drainage area was lower than the median value across all reaches, or “downstream”, otherwise. The choice of median drainage area is warranted, as it enables a fair comparison between location groups constituted by an equal number of sites, and it creates an unbiased split with respect to the catchment studied. For comparability to other catchments (and its common use), we also provided results at the Strahler order level (see Discussion and Supplementary Fig. S8). Within each location group, we picked pairs of flow-unconnected sites such that each site appeared in only one pair; the choice of pairs was operated randomly, and was stopped when no other pair could be formed from the sites that had not been picked yet. Note that we chose to limit our attention to ${\beta}$-diversity of flow-unconnected sites in order to correct for the fact that downstream sites are more likely to be connected by flow than upstream sites (and hence inherently more prone to show similar community compositions), since the latter mostly consist of headwater reaches. Moreover, picking each site only once ensures that measures of ${\beta}$-diversity among all pairs are mutually independent. For each so-obtained pair, Jaccard distance was evaluated via the *betapart* R-package^73^, which also allowed partitioning total ${\beta}$-diversity into nestedness and turnover components, a widely supported approach^74,75^. We accounted for the stochasticity in the choice of pairs by repeating the pair selection process 100 times. The effect of location was deemed significant if the equal-tailed 95% confidence interval of the mean Jaccard distance across one location group did not overlap with that of the other group.

Temporal patterns of ${\beta}$-diversity were evaluated by comparing, within each taxonomic group, predicted presence/absence for all taxa at different seasons via the Jaccard distance evaluated at every reach. In particular, we treated the spring season as a benchmark and focused on patterns of spring-to-summer and spring-to-autumn temporal ${\beta}$-diversity. We chose spring as a benchmark because this is the season in which most taxa were observed (Supplementary Fig. S2), and the season for which biodiversity estimates in riverine systems (in particular for invertebrates) are generally conducted^6^, thus it is a benchmark also in the general context of conservation biology. We then linearly regressed these patterns against drainage area to possibly detect an upstream/downstream gradient on temporal ${\beta}$-diversity. In order to assess the significance of such trends, we adopted the same bootstrapping procedure that was earlier described with respect to *α*-diversity. Moreover, we computed temporal *β*-diversity (expressed as Jaccard distance) for the eDNA data across seasons and taxonomic groups.

Finally, we assessed differences in *α*- and temporal ${\beta}$-diversities between (“predicted”) eDITH model results and (“raw”) eDNA data by comparing the means of the two distributions (values across all river reaches for a given taxonomic group and season) via *t*-tests. We refrained from using non-parametric tests even though the variables analyzed might not follow a normal distribution (specifically, this is the case for Jaccard distances used to assess temporal ${\beta}$-diversity, which are bound between 0 and 1 and often show values close to these boundaries for communities constituted by few taxa): indeed, given that both *α*- and temporal ${\beta}$-diversities can assume discrete values (and importantly, a limited number thereof for groups containing few taxa such as fish), comparing medians as prescribed by non-parametric tests is suboptimal. We also assessed the effect of drainage area on raw patterns of *α*- and temporal *β*-diversities via linear models; in this case, we used *p*-values from the linear model fits to determine significance of these trends, as we assumed that eDNA data were independent from each other. As for spatial ${\beta}$-diversity, we were unable to contrast predicted patterns with raw ones, because only 8 out of 73 eDNA sampling sites fell under the “upstream” category according to the partitioning on median drainage area operated. All analyses were performed in R v.4.2.0.

In our analyses, we opted to use drainage area as the variable defining the spatial position of local communities (reaches) in the river network. Drainage area is the master variable controlling the bulk of hydrological and geomorphological features that shape a fluvial landscape (such as water discharge, velocity, river width and depth^76^) but also organic matter and nutrient availability^77–79^, and hence also determine local habitat conditions. Thus, its use is, both from a hydrological as well as ecological perspective, highly suitable. Importantly, however, increasing *α*- and decreasing ${\beta}$-diversity patterns in river networks had previously been described mostly with respect to stream order^7,9,18^; our choice of using drainage area is consistent with previous studies, as this variable varies predictably with stream order according to Horton’s law on drainage areas^80^. It is likely, however, that our approach might have an impact on estimation of trends of spatial ${\beta}$-diversity. The partitioning of reaches into an “upstream” and a “downstream” group was roughly equivalent to comparing headwaters (i.e. reaches with stream order equal to 1) to all other reaches (Supplementary Fig. S8). While pairwise Jaccard distances for reaches of stream order 4 or 5 could be lower with respect to those for more upstream reaches, it is unfeasible to statistically assess the magnitude of this trend for the different stream order values, because of the paucity of reaches with stream order $\ge 4$ in the river network, and the fact that these tend to be connected by flow (which is likely to bias conclusions on ${\beta}$-diversity, as discussed above). Note also that the predominance of headwaters with respect to reaches of high stream order is not limited to the catchment studied here, but is rather a universal feature of river networks^81^.

## Supplementary Information

Supplementary Information.

## Data Availability

The datasets analysed during the current study are available in the Github repository https://github.com/lucarraro/eDITH_RiverDNA. A permanent link will be created upon manuscript acceptance.
