# Supplementary material for: Modelling environmental DNA transport in rivers reveals highly resolved spatio-temporal biodiversity patterns
Source: Sci Rep. 2023 May 31;13:8854. doi: 10.1038/s41598-023-35614-6 (PMC10232434; doi:10.1038/s41598-023-35614-6)
Supplement: Supplementary file 1 — Supplementary Information. [file 41598_2023_35614_MOESM1_ESM.pdf]

Supplementary Information: Modelling environmental DNA transport in rivers reveals highly resolved spatio-temporal biodiversity patterns

Luca Carraro[1,2,*], Rosetta C. Blackman[1,2], Florian Altermatt[1,2]

[1] *Department of Evolutionary Biology and Environmental Studies, University of Zurich, Zürich, Switzerland*

[2] *Department of Aquatic Ecology, Swiss Federal Institute of Aquatic Science and Technology, Eawag, Dübendorf, Switzerland*

[*] *Corresponding author: luca.carraro@eawag.ch*

Contents

Supplementary Tables **2**

Supplementary Figures **7**

Supplementary Tables

Table S1: Mean values of pairwise spatial β-diversity (calculated among all 1908*1907/2 pairs of sites). β_{JAC}: Jaccard distance; β_{JTU}/β_{JAC}: proportion of Jaccard distance explained by the turnover component. Partitioning of β-diversity is calculated via the *betapart* package [1].

	β_{JAC}			β_{JTU}/β_{JAC}		
	spring	summer	autumn	spring	summer	autumn
Fish	0.501	0.527	0.591	0.335	0.358	0.500
Invert.	0.752	0.772	0.764	0.823	0.853	0.803
Bacteria	0.499	0.635	0.560	0.753	0.805	0.711

Table S2: Mean values of predicted and raw temporal β-diversity. β_{JAC}: Jaccard distance; β_{JTU}/β_{JAC}: proportion of Jaccard distance explained by the turnover component. Partitioning of β-diversity is calculated via the *betapart* package [1].

		β_{JAC}		β_{JTU}/β_{JAC}	
		spring-summer	spring-autumn	spring-summer	spring-autumn
Predicted	Fish	0.562	0.360	0.266	0.255
	Invert.	0.754	0.742	0.866	0.777
	Bacteria	0.694	0.659	0.831	0.909
Raw	Fish	0.527	0.221	0.207	0.107
	Invert.	0.781	0.731	0.782	0.670
	Bacteria	0.631	0.487	0.656	0.771

Table S3: List of genera detected.

**Fish**					
1	Barbatula	Cottus	Gobio	Phoxinus	Scardinius
2	Barbus	Cyprinus	Perca	Salmo	Squalius
3	Carassius	Esox			
**Invertebrates**					
1	Acanthocyclops	Cyclops	Gammarus	Lumbricillus	Pristina
2	Amphinemura	Cypridopsis	Gyrodactylus	Melampophylax	Prodiamesa
3	Anopheles	Dero	Glossosoma	Micropsectra	Prosimulium
4	Antocha	Diamesa	Habroleptoides	Nemoura	Protonemura
5	Asellus	Dicranomyia	Habrophlebia	Ophidonais	Rhyacophila
6	Baetis	Ecdyonurus	Hydra	Orthocladius	Rhithrogena

7	Caenis	Eiseniella	Hydropsyche	Paratrichocladius	Riolus
8	Capnioneura	Elmis	Hydroptila	Perla	Sericostoma
9	Chaetogaster	Elodes	Isohypsibius	Perlodes	Serratella
10	Chaetonotus	Epeorus	Isoperla	Philopotamus	Simulium
11	Chironomus	Ephemera	Keratella	Plumatella	Stenostomum
12	Chydorus	Ephemerella	Leuctra	Polyarthra	Stylodrilus
13	Cloeon	Ephydatia	Limnephilus	Polypedilum	Takobia
14	Conchapelopia	Euchlanis	Limnius	Potamophylax	Tanytarsus
15	Craspedacusta	Eucyclops	Limnodrilus	Potamopyrgus	Thermocyclops
16	Cricotopus	Eukiefferiella	Limnophyes	Potamothrix	Tvetenia
**Bacteria**					
1	12up	Comamonas	Hydrogenophaga	Parafilimonas	Sandaracinus
2	Acetobacteroides	Corynebacterium_1	Hymenobacter	Parasediminibacterium	Sandarakinorhabdus
3	Acholeplasma	Coxiella bacter	Hyphomicrobium	Parasegeti-	Sarcina
4	Achromobacter	Crenothrix	Iamia	Pedobacter	Schlesneria
5	Acidibacter	Crocinitomix	Ilumatobacter	Pedomicrobium	Sediminibacterium
6	Acidiphilium	Cytophaga	Iodobacter	Pelosinus	Serratia
7	Acidovorax	Deefgea	Kurthia	Peredibacter	Sideroxydans
8	Acinetobacter	Deinococcus	Lachnoclostridium	Perlucidibaca	Silanimonas
9	Actinoplanes	Delftia	Lacibacter	Petrimonas	Simiduia
10	Aeromonas	Devosia	Lactobacillus	Phacotus_lenticularis	Simplicispira
11	Aetherobacter	Dickeya	Lactococcus	Phaeodactylibacter	SM1A02
12	AKYG587	Dietzia	Leadbetterella	Phascolarctobacterium	Snowella
13	Algoriphagus	Dinghuibacter	Leeia	Phaselicystis	Solitalea
14	Alistipes	Dongia	Legionella	Phenylobacterium	Sorangium
15	Alkanindiges	Duganella	Leptolyngbya	Phormidium	Sphaerochaeta
16	Alloprevotella	Dyadobacter	Leptothrix	Phycisphaera	Sphaerotilus
17	Altererythrobacter	Dysgonomonas	Leptotrichia	Pir4_lineage	Sphingobacterium
18	Anaerocella	Elstera	Leucobacter	Pirellula	Sphingobium

19	Aquabacterium	Emticicia	Limnobacter	Planctomyces	Sphingomonas
20	Aquaspirillum	Enhydrobacter	Limnohabitans	Planktothrix	Sphingopyxis
21	Aquicella	Erysipelothrix	Luteimonas	Pleurocapsa	Sphingorhabdus
22	Aquimonas	Euglenaria_anabaena	Luteolibacter	Polaromonas	Spirochaeta_2
23	Arcicella	Exiguobacterium	Lysobacter	Polyangium	Spirosoma
24	Arcobacter	Facklamia	Macellibacteroides	Polymorphobacter	Sporichthya
25	Arenimonas	Faecalibacterium	Magnetospirillum	Polynucleobacter	Staphylococcus
26	Armatimonas	Fastidiosipila	Marinospirillum	Prevotella_9	Stenotrophobacter
27	Asaia	Ferruginibacter	Massilia	Propionivibrio	Stenotrophomonas
28	Asticcacaulis	Fibrella	Megamonas	Prosthecobacter	Steroidobacter
29	Azospirillum	Filimonas	Meganema	Proteiniclasticum	Streptococcus
30	Bacillus	Flavihumibacter	Merismopedia	Proteiniphilum	Streptomyces
31	Bacteriovorax	Flavitalea	Mesorhizobium	Pseudarcicella	Sulfurifustis
32	Bacteroides	Flavobacterium	Methanogenium	Pseudendoclonium_akinetum	Sulfuritalea
33	Bauldia	Flectobacillus	Methylobacterium	Pseudohongiella	Sulfurospirillum
34	Bdellovibrio	Fluviicoccus	Methyloparacoccus	Pseudomonas	Sutterella
35	Beggiatoa	Fluviicola	Methylophilus	Pseudorhodoferax	Synechococcus
36	Bifidobacterium	Fluviimonas	Methylotenera	Pseudoxanthomonas	Taibaiella
37	Blastocatella	Fodinicola	Microbacterium	Psychrobacter	Terrimicrobium
38	Blautia	Fusibacter	Microcoleus	Reyranella	Terrimonas
39	Bosea	Fusobacterium	Mucilaginibacter	Rheinheimera	Thauera
40	Brevundimonas	Gaiella	Mycobacterium	Rhizobacter	Thermomonas
41	Bryobacter	Gemmata	Nakamurella	Rhizobium	Thiothrix
42	BSV13	Gemmatimonas	Nannocystis	Rhizomicrobium	Tissierella
43	Catenibacterium	Geobacter	Nevskia	Rhizorhapis	Treponema_2
44	Caulobacter	GKS98_fresh-	Nitrospira	Rhodobacter	Trichococcus

		water-_group			
45	Cellvibrio	Granulicella	Niveispirillum	Rhodococcus	Truepera
46	Chamaesiphon	H16	Nocardioides	Rhodoferax	Turicibacter
47	Chitinibacter	Haematospirillum	Novosphingobium	Rhodomicrobium	Uliginosibacterium
48	Chitinimonas	Haliangium	Oleiphilus	Rickettsia	Undibacterium
49	Chitinivorax	Haliea	Oligoflexus	Rickettsiella	Variibacter
50	Chitinophaga	Halioglobus	Oligosphaera	Roseiflexus	Verrucomicrobium
51	Chroococcidiopsis	Haliscomenobacter	OM27_clade	Roseococcus	Vogesella
52	Chryseobacterium	Haloferula	Opitutus	Roseomonas	Woodsholea
53	Chryseolinea	Halomonas	Paenibacillus	Rubellimicrobium	Yersinia
54	Chthoniobacter	Herpetosiphon	Paludibacter	Rudanella	Z20
55	Cloacibacterium	hgcI_clade	Paludibaculum	Runella	Zoogloea
56	Clostridium	Hirschia	Parabacteroides	Sandaracinobacter	Zymomonas
57	Collinsella	Holdemanella			

Table S4: List of land cover, geological, and morphological covariates used (from Carraro et al. [3]). Geographical covariates are reproduced in Fig. S3.

Type	Acronym	Covariate	Extent
Land cover	L-FO	Forest	Local
Land cover	L-RO	Rocks	Local
Land cover	L-UR	Urban area	Local
Land cover	L-OR	Orchard	Local
Land cover	L-SW	Swamp	Local
Land cover	L-LA	Lake	Local
Geological	G-AL	Alluvial rocks	Upstream
Geological	G-MO	Moraines	Upstream
Geological	G-AP	Alpine sediments	Upstream
Geological	G-WA	Superficial waters	Upstream
Geological	G-LO	Loess	Upstream
Geological	G-SC	Scree	Upstream
Geological	G-PE	Peat	Upstream
Morphological	M-US	Mean upstream channel slope	Upstream
Morphological	M-DA	Drainage area	Upstream
Morphological	M-LS	Local channel slope	Local
Morphological	M-LE	Local elevation	Local
Morphological	M-SO	Stream order	Local

Supplementary Figures

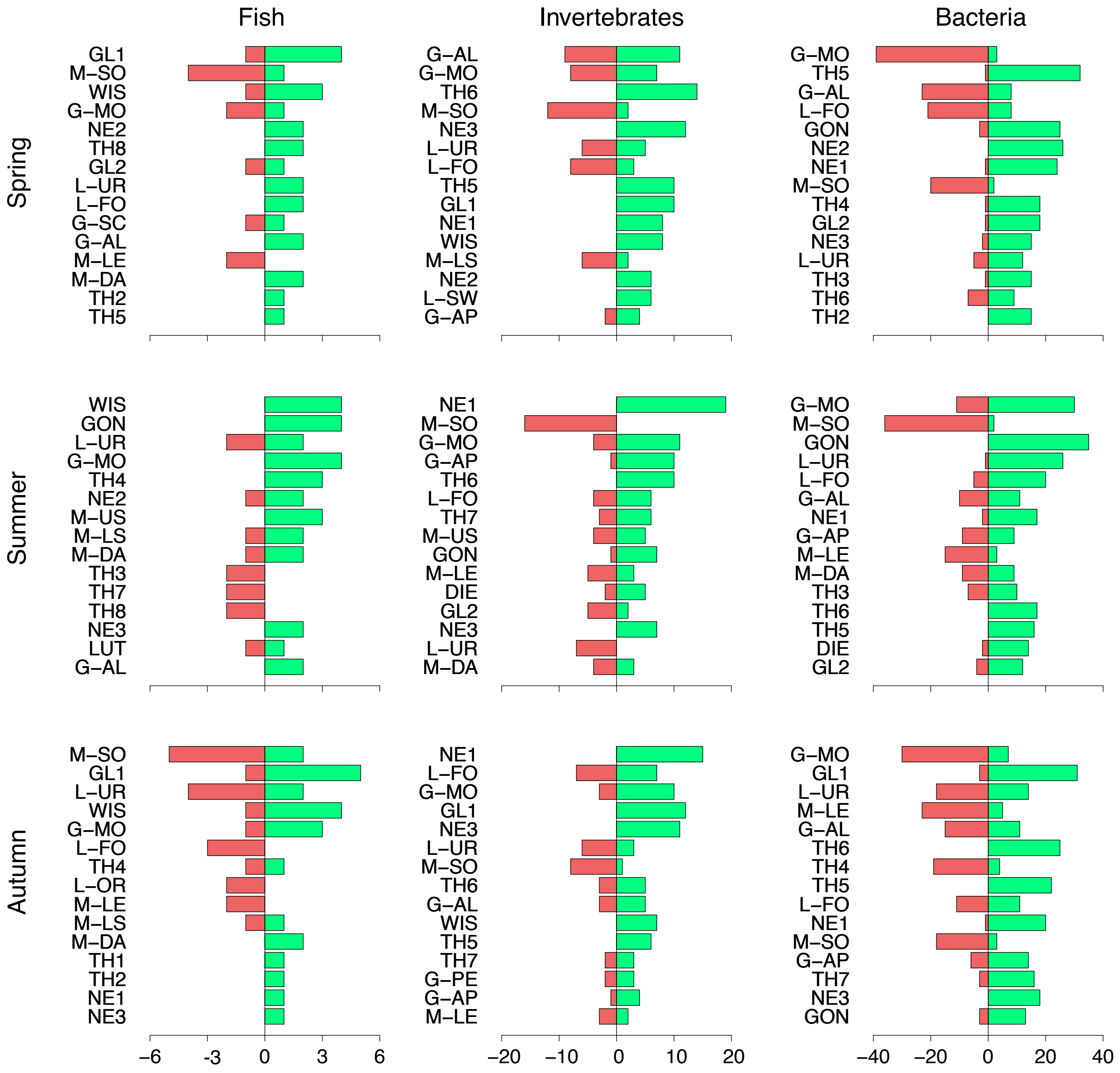

Figure S1: Significance of environmental covariates. The x-axis expresses the number of genera within a given taxonomic group and season for which a significant positive (green) or negative (red) effect was predicted by the eDITH model. Acronyms in the y-axis identify environmental covariates (see Table S4 and Fig. S3 for the key). For graphical reasons, only the 15 (out of 35) most significant variables are shown. Positive (negative) significance is attributed if the 2.5th-97.5th percentile range of the posterior distribution of the respective β parameter was positive (negative). See Carraro et al. [3] for details.

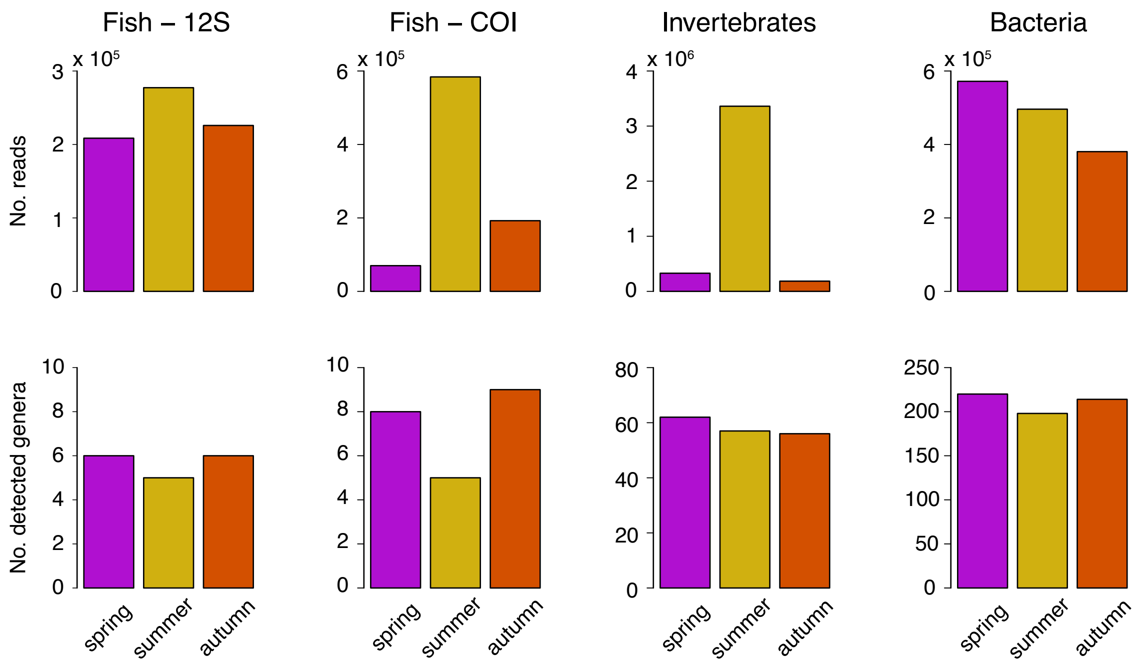

Figure S2: Summary of eDNA metabarcoding data pooled over sampling sites, and partitioned by taxonomic group, barcode region (for fish), and season. Note that here the data corresponding to fish genera *Barbus*, *Gobio* and *Phoxinus* are included in the histograms for both 12S and COI barcode regions.

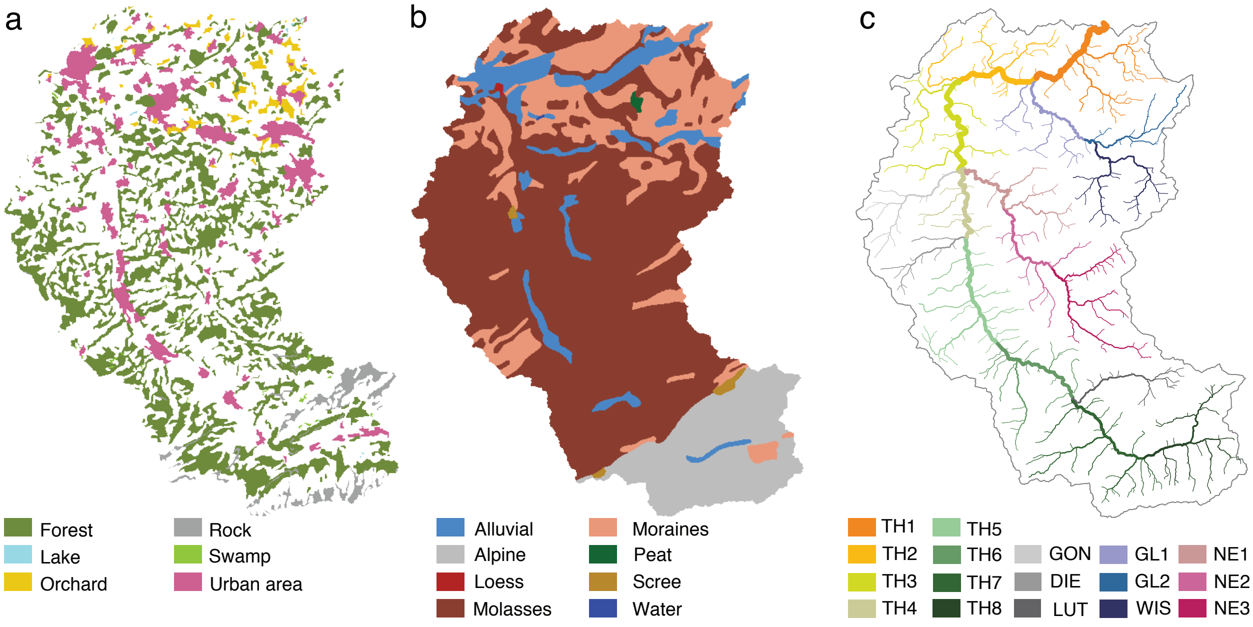

Figure S3: Thematic maps of the Thur catchment: a) land cover, b) geology, c) geographical covariates (from Carraro et al. [3]). Maps were generated via the *rivnet* R-package [2].

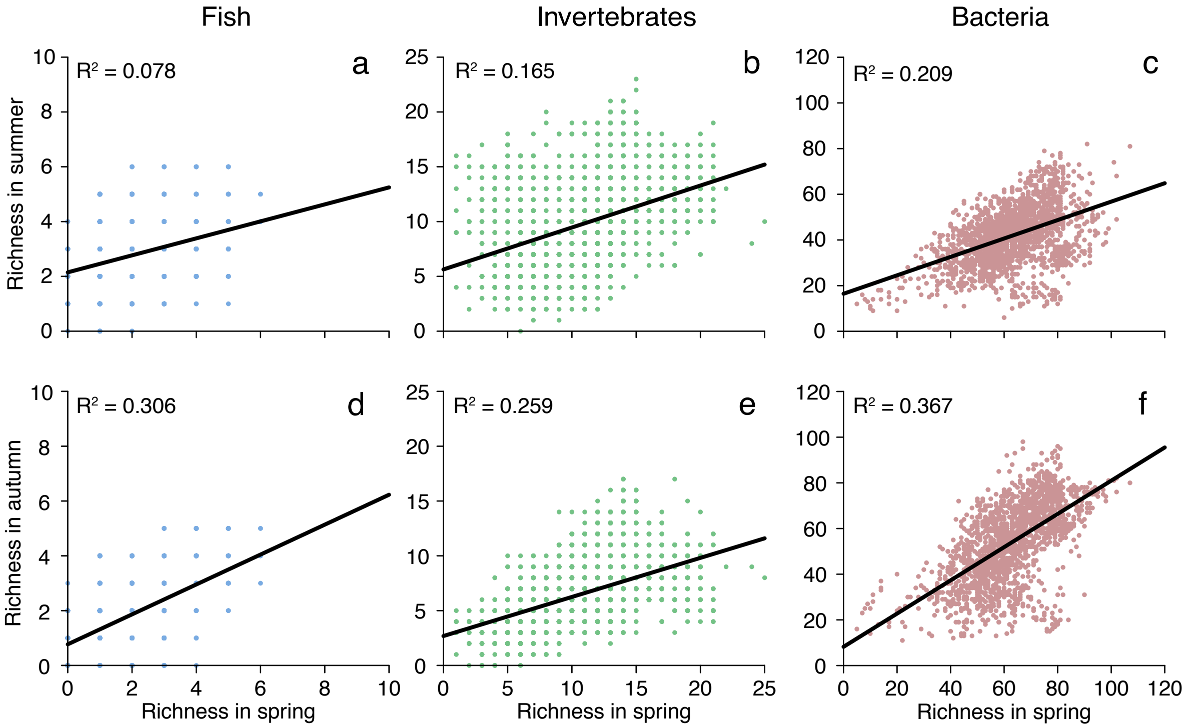

Figure S4: Relationship between patterns of α-diversity with respect to different seasons (top row: richness in spring vs. richness in summer; bottom row: richness in spring vs. richness in autumn. Black solid lines represent linear regressions. R^2 values are reported on the top-left corner.

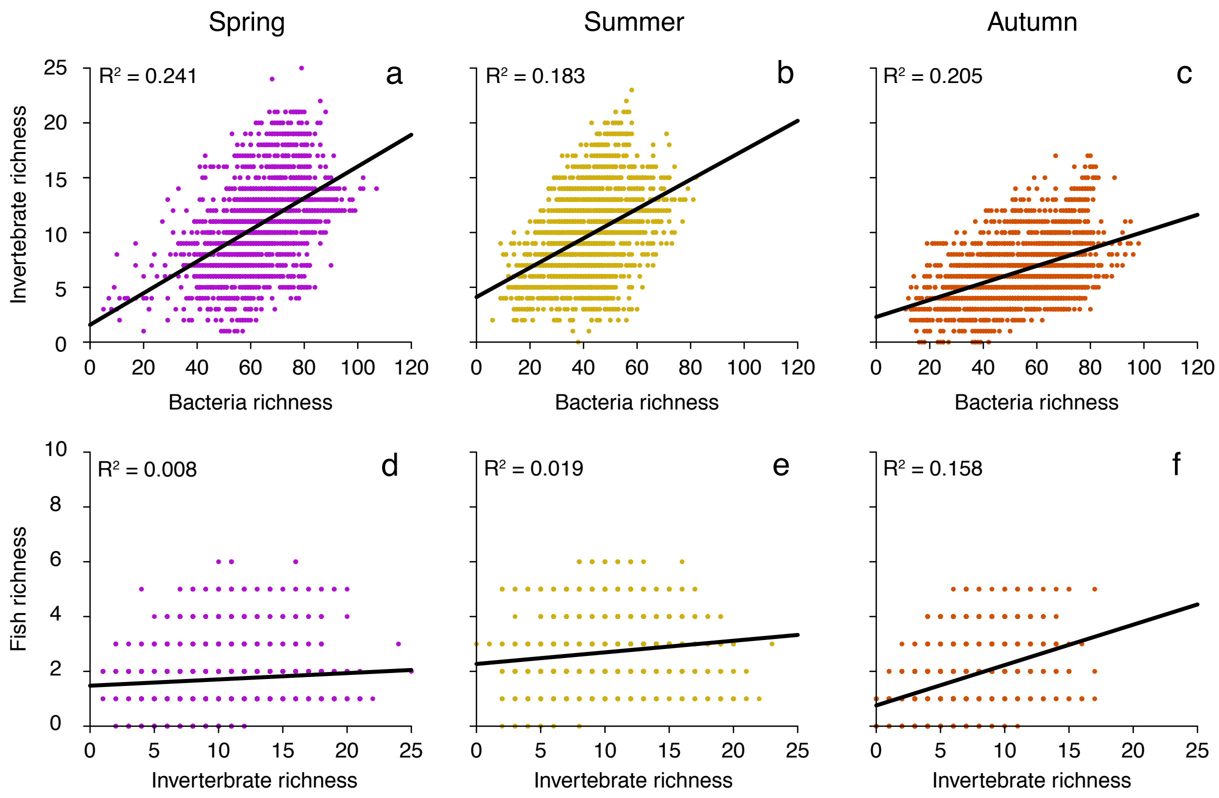

Figure S5: Relationship between patterns of α-diversity with respect to different taxonomic groups (top row: bacteria richness vs. invertebrate richness; bottom row: invertebrate richness vs. fish richness). Black solid lines represent linear regressions. R^2 values are reported on the top-left corner.

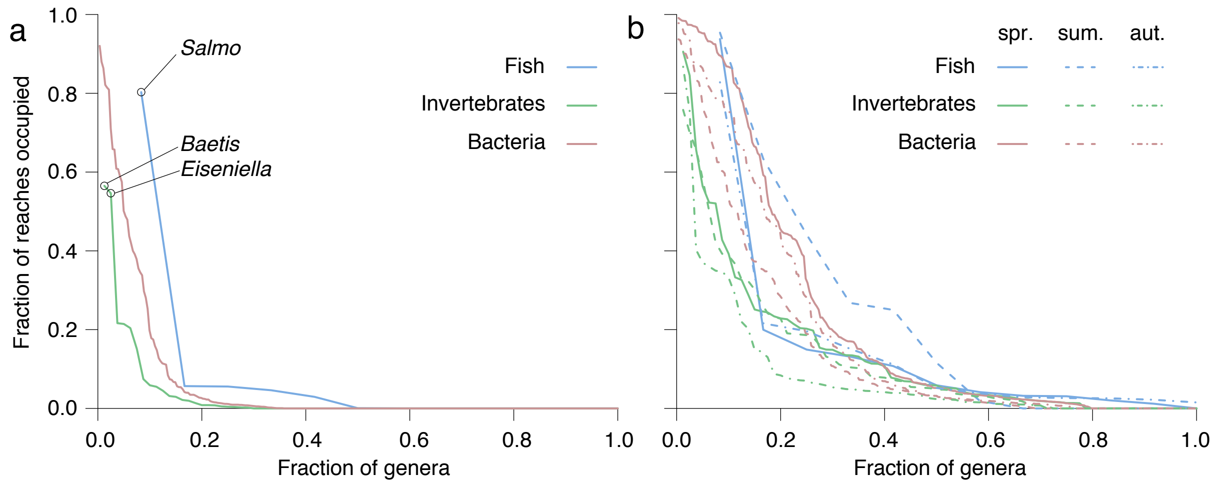

Figure S6: Distribution of genera for a given taxonomic group sorted by the fraction of reaches occupied as predicted by the eDITH model. a) Values pooled across seasons (here a taxon is considered present in a reach only if presence therein was predicted at all three seasons); b) values partitioned by season. Interpretation: a point (x, y) on a curve expresses that there is a fraction x of genera that are predicted to occupy a fraction $\geq y$ of reaches. In panel a, the most abundant fish and invertebrate genera are marked with dots.

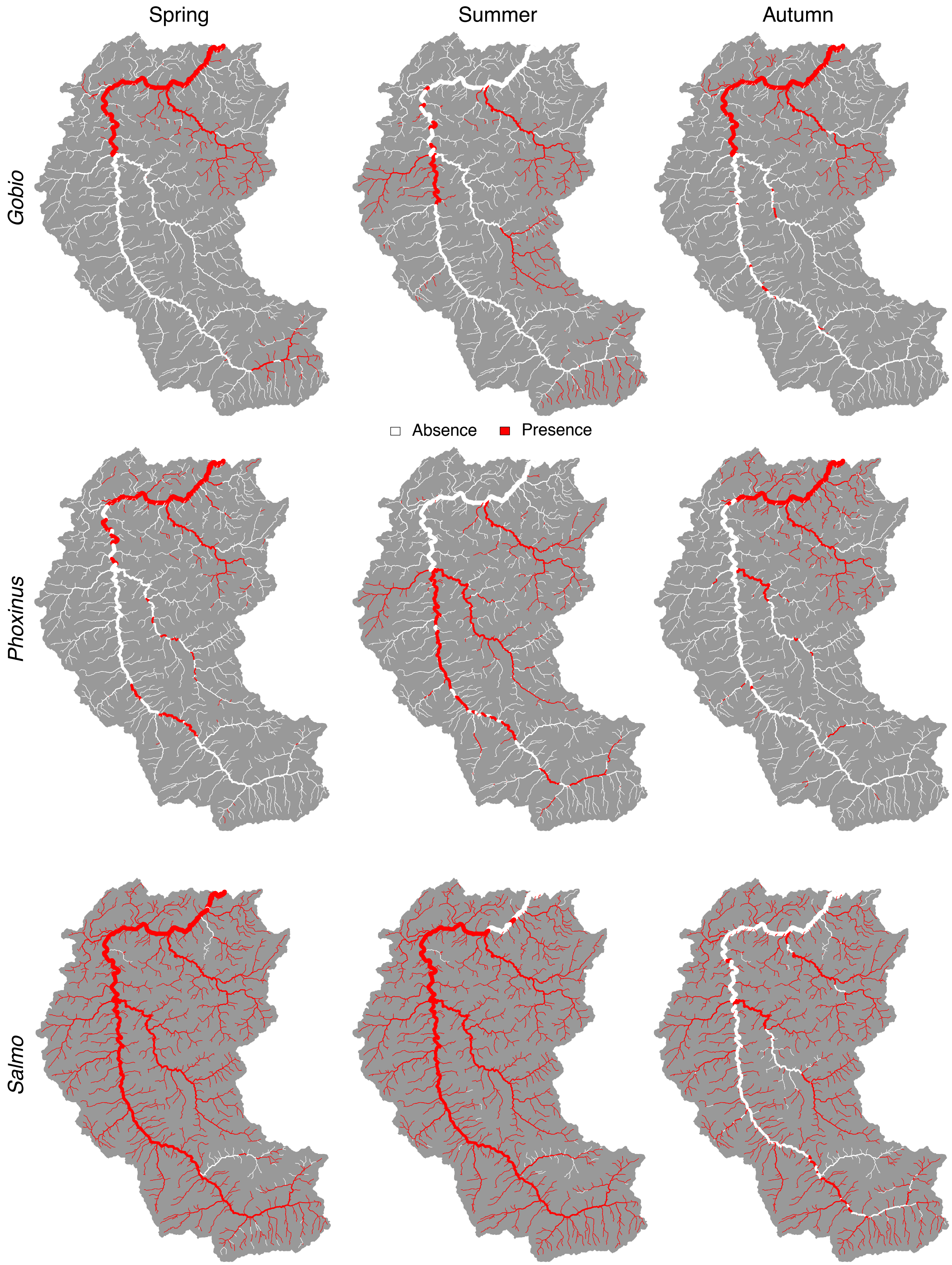

Figure S7: Presence-absence maps as predicted by the eDITH model for fish genera *Gobio*, *Phoxinus* and *Salmo* across the different seasons. Maps were generated via the *rivnet* R-package [2].

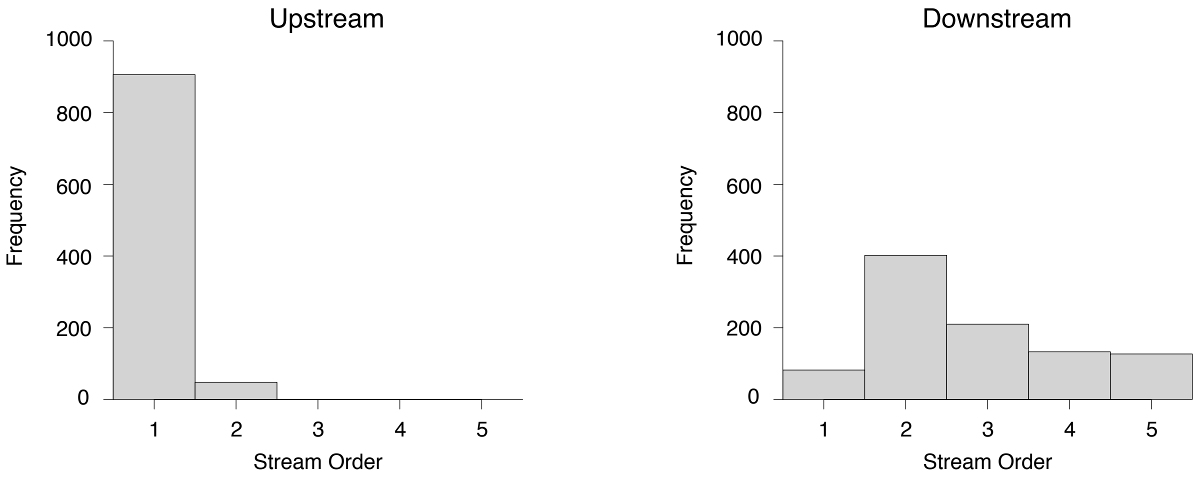

Figure S8: Distribution of stream order values within the groups used for analysis of spatial β-diversity patterns. ”Upstream” (”Downstream”) refers to reaches whose drainage area was lower (higher) than the median.

References

[1] Baselga, A. and Orme, C. D. L. (2012). betapart: an R package for the study of beta diversity. *Methods in Ecology and Evolution*, 3(5):808–812.

[2] Carraro, L. (2023). Seamless extraction and analysis of river networks in R: The rivnet package. *SSRN*. doi:10.2139/ssrn.4395305.

[3] Carraro, L., Mächler, E., Wüthrich, R., and Altermatt, F. (2020). Environmental DNA allows upscaling spatial patterns of biodiversity in freshwater ecosystems. *Nature Communications*, 11(1):3585.
